# Shape dependence of the release rate of chemicals from plastic microparticles

**DOI:** 10.1007/s11356-022-21440-2

**Published:** 2022-07-12

**Authors:** Riccardo Frazzetto, Diego Frezzato

**Affiliations:** Department of Chemical Sciences, University of Padova, via Marzolo 1, I-35131 Padova, Italy

**Keywords:** Microplastics, Release of chemical additives from microparticles, Release of contaminants in aquatic environments, Shape-dependent release from microparticles, Diffusion in rubbery microplastics

## Abstract

The release of chemical additives from plastic microparticles in the aqueous phase represents a potential indirect threat for environment and biota. The estimate of the release timescale is demanded for drawing sensible conclusions on quantitative grounds. While the microparticles are generally taken to be spherical for ease of modelling, in reality the variety of shapes is large. Here, we face the problem of working out an empirical simple expression for estimating the release times for arbitrary shapes, assuming that the plastic material is in the rubbery state, that the dynamics inside the particle is a diffusion process, and that the release is irreversible. Our inspection is based on numerical simulations of the release process for randomly generated instances of regular and irregular geometries. The expression that we obtain allows one to estimate the release time in terms of the corresponding time (easy to compute) for the equal-volume spherical particle taken as reference, and of the ratio between the surface areas of particle and equivalent sphere.

## Introduction

Micro-sized plastic debris are widespread in all environmental compartments on a planetary scale and are cause of concern for various reasons, including direct and indirect threats for the health of all forms of biota (Teuten et al. 2009; Kane and Clare 2019; Khalid et al. 2020; Ge et al. 2021). Regarding the indirect threats in aquatic environments, microplastics have been early addressed as possible vectors of pollutants^1^ and as source of toxic additives released in the hosting medium (Mato et al. 2001; Teuten et al. 2007; Amelia et al. 2021), a matter still under intense study and debate (Koelmans et al. 2016; Alimi et al. 2018). Among the additives, we mention flame retardants, plasticizers, additives for heat and UV resistance, colorants, and other chemicals added during the manufactoring process for conferring specific properties to the material (Fred-Ahmadu et al. 2020). The quantitative characterization of the transfer between plastic and medium lies at the core of any model for the distribution of the chemicals among the phases, and even for the transport on the wider scale.

Here, we focus on the irreversible release of additives from plastic microparticles into the aqueous medium, with the aim of inspecting how the rate of release does scale with respect to the area of the exposed surface at a fixed particle’s volume. We adopt a statistical approach based on the inspection, through simulations and global analysis of the outcomes, of large sets of shapes for several types of geometries. First, let us fix the physical framework at the basis of our modelling.

We shall refer to amorphous plastic materials in the rubbery state above the glass transition temperature.^2^ This is the representative situation (Town and van Leeuwen 2020) to which one commonly refers when facing the release from plastic materials in the aquatic environment. This choice is supported by considering the main types of plastic produced on the planetary scale. An estimate on the main common types of plastics ever made up to 2015 was done in ref. Geyer et al. (2017). We can deduce that about 50% of plastics is constituted by polymeric materials that, at ambient temperature, are in the rubbery state. The main plastics of such a kind are polyethylene (low-density, high-density, linear low-density) and polypropylene. Direct inspections have revealed that polyethylene and polypropylene are the predominant plastics also in aquatic environments (Isobe et al. 2014; Enders et al. 2015; Frère et al. 2017). Thus, we may take the rubbery state as a representative situation for our analysis. Under such assumption, the motions of the chemical species inside the particle can be modelled as a diffusive process induced by the thermal fluctuations of flexible portions of the polymeric structure. For simplicity, we assume the material homogeneous, and take the diffusion coefficient *D* as constant. It is supposed that *D* is known, for the given species in the given material, either from direct experimental assessment or from semi-empirical relations. For instance, for a class of brominated flame retardands, *D* could be related with the molecular diameter and with the polymer’s glass transition temperature (Sun et al. 2019). Yet, diffusion coefficients in polyethylene have been related with the molar volume of the diffusing species (Lohmann 2012).

With the above positions, the release can be modelled as diffusion inside the material with irreversible escape through the exposed surface (i.e., by treating the interface plastic-medium as an absorbing boundary). A simple but likely assumption is to consider initial homogeneous volumetric concentration $c_0$. In addition, the external concentration is considered to be vanishingly small even close to the interface, so that the assumption of irreversible leakage is licit. This is acceptable if the medium is well stirred in the neighborhood of the particle; otherwise, a different kind of modelling should be adopted (Endo et al. 2013).

Let $p_\text {int}(t)$ be the fraction of chemical species still present inside the particle at the time *t*, i.e., $p_\text {int}(t) = m_\text {int}(t) / (c_0 V)$ being *V* the volume of the particle. The temporal profile of $p_\text {int}(t)$ starts from 1 and monotonically decays to 0. Commonly adopted descriptors of such a decay are the times $\tau _\alpha$ at which

1 $$\begin{aligned} p_\text {int}(\tau _\alpha ) = 1-\alpha \end{aligned}$$

In particular, $\tau _{0.5}$ is the common half-life time, while $\tau _{0.95}$ can be conventionally taken as the time required for achieving, in practice, the complete release (Endo et al. 2013). Under the above assumptions, the geometry typically considered is that of a spherical particle of radius $r_s$. For such a geometry, the evolution of the internal concentration profile is analytically known, the mass flux at the surface can be exactly evaluated, and the internal fraction $p_\text {int}^s(t)$ (throughout, the superscript “s” stands for sphere) is given by the well-known Crank’s expression (Crank 1975):

2 $$\begin{aligned} p_\text {int}^s(t) = \frac{6}{\pi ^2} \sum _{n=1}^{\infty } n^{-2} \, e^{-n^2 \pi ^2 D t / r_s^2} \end{aligned}$$

In combination with Eq. 1, this expression allows one to determine the $\tau _\alpha ^s$ numerically. Equation 2, or variants in which an effective diffusion coefficient is employed to take into account speciation reactions (Town and van Leeuwen 2020), is frequently used for a first-level study of the release process from microplastics.

A question arises: How are the $\tau _\alpha$ affected by a change of the microparticle’s shape in passing from spherical to other geometries? It is known that microplastics, especially when formed by fragmentation processes (Wayman and Niemann 2021), can assume a variety of shapes including spheres, fibers, flakes, and generally irregular fragments (Paul-Pont et al. 2018). In particular, fibers (filaments and lines) are abundant in the acquatic enviroments and may derive directly either from fishing lines and nets or from degradation of primary plastics (Kane and Clare 2019; Free et al. 2014). For instance, in a study conducted in the East China Sea it was found that fibers are the dominant morphotype, followed by granules and films (Zhao et al. 2014). Intuitively, one may expect that for non-spherical shapes the release times are shorter because, at fixed volume, the surface area is higher. Such shortened release times might be a priori a cause of concern; hence, a more reliable estimate is demanded. The *exact* quantitative answer to the above question must be clearly given case by case. In spite of such case-dependent specificity, one may ask if there might be an empirical relation to link $\tau _\alpha$ with some suitable coarse descriptor (to be found) quantifying the deviation from the spherical shape at a fixed volume of the particle.

In this work, we face such a problem by combining intuitive expectation and numerical simulations for some classes of regular and irregular geometries. For each kind of geometry, a large number of instances is generated at random by varying the geometric parameters. For each instance, solving numerically the diffusion equation is a hard task since it requires the discretization of the internal volume of the particle and the enforcing of the absorbing boundary conditions on the exposed surface. As the particle’s shape becomes locally very featured, this requires employing an irregular and case-dependent tri-dimensional grid. In this work, we propose a new approach to by-pass the cumbersome numerical solution of the diffusion equation. The strategy consists in producing a large number of Brownian trajectories starting from points randomly generated in a uniform way inside the particle. Each moving point represents a molecule whose trajectory is stopped when the delimiting surface is crossed. From the statistical ensemble of trajectories, we get the time evolution of the survival fraction $p_\text {int}(t)$, and hence the release times $\tau _\alpha$ for chosen values of $\alpha$. The global synthesis of the outcomes then allows us to work out the key result, namely the empirical relation Eq. 3 presented later. A similar kind of strategy, i.e., making a systematic exploration as extensive as possible, is nowadays adopted in several contexts of physical statistics to make conjectures and discover new laws before formal proofs, and has been recently applied to the assessment of the release of chemicals from materials under intermittent conditions (Frezzato et al. 2022). To the best of our knowledge, the present strategy has not yet been employed to the release of chemical species from plastic microparticles. The results are illustrated in the next section, while the details of the methodology are provided in “Computational details.”^3^

The main finding is that the ratio $A/A_s$, where *A* is the surface area of the given particle and $A_s$ is the area of the equal-volume sphere, suffices to connect $\tau _\alpha$ with the reference $\tau _\alpha ^s$ easily obtainable from Eq. 2. The key outcome is Eq. 3 given later. Although such an expression is nothing but a tentative empirical law to be used for a rough estimation of $\tau _\alpha$, it might be relevant for getting a more reliable estimate of the leakage timescale. For instance, even the sole indication that $\tau _{0.5}$ might be 2–3 times shorter than the value expected for the spherical shape can be a useful information.

## Empirical results

As outlined in the “Introduction,” we empirically face the problem by means of numerical simulations done for particles of several shapes. The generation of a large ensemble of Brownian trajectories starting from internal points drawn at random in an unbiased way allows us to achieve $p_\text {int}(t)$ and, hence, to get the release times $\tau _\alpha$. Details about the method are provided in the section “Computational details.” For each particle’s shape, we also consider the associate sphere of equal volume and determine the times $\tau _\alpha ^s$ from $p_\text {int}^s(t)$ in Eq. 2.

Both regular and irregular geometries have been considered. The regular geometries are as follows: the parallelepiped with sides of length *a*, *b*, and *c* (ranging from square slab to elongated parallelepiped); the ellipsoid with semi-axes of length $a=b$ and *c* (from lens-like to needle-like shape); the torus generated by the revolution of the circle of radius *a* with revolution radius $R_0 \ge a$ (from doughnut-like to ring-like shape); multi-bead structures made of a number of beads (up to 100) of radii generated at random. The choice of such geometries was motivated not only by the ease of checking on the fly when the moving point crossed the delimiting surface, but also by the fact that such geometries capture some essential features of real plastic microparticles. The toroidal shape, a simple example of non-convex shape, mimics an object with a hole. The multi-bead structure is chosen since it corresponds to a variety of structures in which the small beads may be connected one with the others in several ways: from pearl-necklace–like structures to irregular agglomerates. The transit between the beads is meant to occur at single points of contact; hence, the multi-bead structure is an extreme case of particles in which different parts are connected by tiny bottlenecks of vanishingly small volumes. The aspect of such regular geometries has been varied by exploring a wide range of values of their parameters (see “Computational details”). For the parallelepiped and the ellipsoidal shapes, the maximum allowed aspect ratio (maximum over minimum linear extension) was 100; the toroidal shapes could range from a doughnut-like shape without hole to a thin ring-like shape with ratio 100 between radius of the ring and radius of the circular cross section; in the multi-bead structures, the radii of the beads varied within a factor 10. The irregular geometries, described later in detail, have been generated by adopting spherical coordinates for describing the delimiting surface, by using a parametric functional form for the surface, and then drawing at random the values of the required parameters. In this case, the geometries are all of convex type for the ease of checking when a trajectory crosses the surface.

**Fig. 1:**
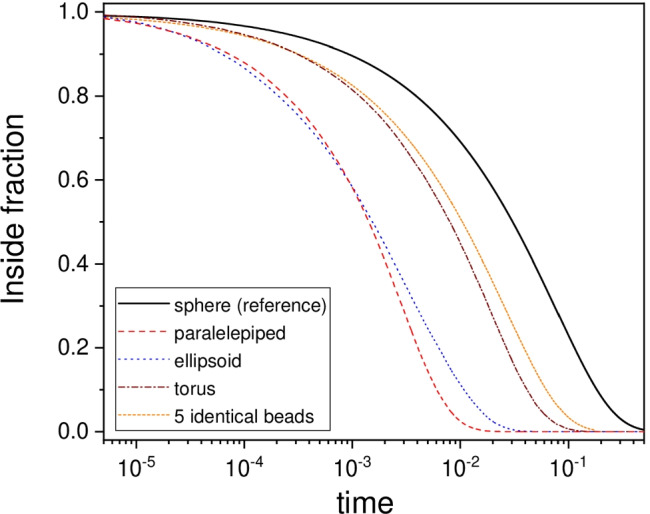
Time dependence of the survival fraction of chemical species inside the particle. The profiles refer to particles of different geometry but equal volume: reference sphere with $r_s = 1$; square slab with $a = b = 5$ and $c = 0.168$ ($A/A_s = 4.2$); ellipsoid with $a = b = 0.2$ and $c = 25$ ($A/A_s = 3.9$); torus with $a = 0.35$ and $R_0 = 1.732$ ($A/A_s = 1.9$); five identical beads with $r_i = 0.585$ ($A/A_s = 1.7$). Physical units of length and time are here irrelevant and are left implicit. The diffusion coefficient *D* was set equal to 1 with reference to such units

Let us begin with the outcomes for the regular geometries. Figure 1 shows the profiles of $p_\text {int}^s(t)$ and $p_\text {int}(t)$ for some instances of the regular geometries at fixed volume. It can be seen how the spherical shape is the one for which the profile lies above all others; hence, the release process is the slowest. This is intuitively expected because, at a fixed volume, the sphere has the smallest surface area. This suggests to consider, at least provisionally, the ratio $A/A_s$ as a potentially relevant geometrical descriptor for expressing the deviation of $\tau _\alpha$ from $\tau _\alpha ^s$.

**Fig. 2:**
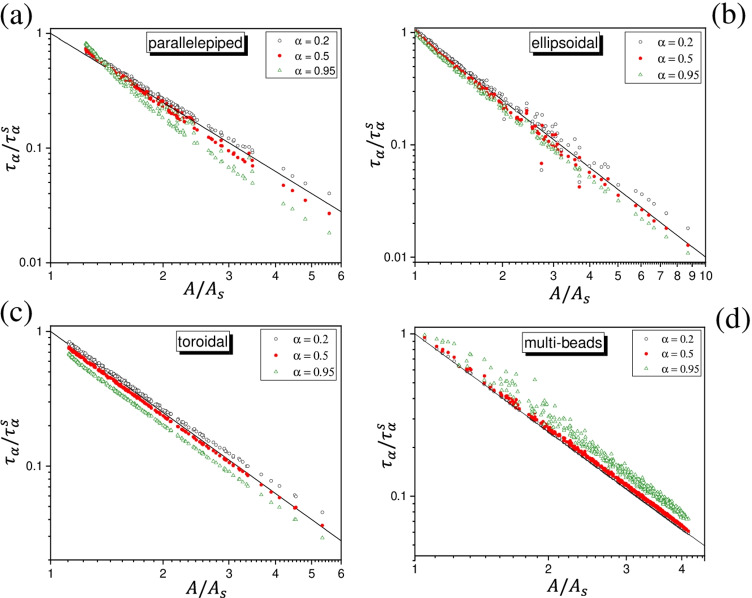
Spread of the ratio $\tau _\alpha /\tau _\alpha ^s$ versus $A/A_s$ (at fixed volume of the particle) for randomly generated instances of several geometries; see the text for details. Open black circles refer to $\alpha = 0.2$ (80% of species still present inside the particle), full red circles to $\alpha = 0.5$ (50% inside), open green triangles to $\alpha = 0.95$ (5% inside). The solid line has slope $-2$ in the double-logarithmic scale here adopted

Figure 2 shows the values of $\tau _\alpha / \tau _\alpha ^s$ against $A/A_s$ for the regular geometries here considered. Each panel shows the data for a number of randomly generated instances (see “Computational details”). The different symbols refer to specific stages of the release: $\alpha = 0.2$ (black open circles), $\alpha = 0.5$ (red full circles) and $\alpha = 0.95$ (green open triangles). The continuous straight line has slope $-2$ in the adopted double-logarithmic scale. What emerges is that, for all $\alpha$, the points are concentrated close to the straight line. This suggests to propose the following empirical relation linking $\tau _\alpha$ with the reference $\tau _\alpha ^s$ through the factor $A/A_s$:

3 $$\begin{aligned} \tau _\alpha \simeq \tau _\alpha ^s \, \left( \frac{A}{A_s} \right) ^{-2} \end{aligned}$$

In this expression, the symbol “$\simeq$” must be interpreted as a “rough but likely estimate of the order of magnitude” of $\tau _\alpha$ without using any other subtler descriptor of the particle’s geometry. We see in fact that the points are distributed around the straight line, and in some cases, depending on $\alpha$, they tend to lay below or above the line. The multi-bead case reveals an interesting behavior. For beads of different radii, each bead has its own release kinetics and the overall decay of $p_\text {int}(t)$ corresponds to a weighted average of the individual decay profiles (see “Computational details”). The distribution of the points in Fig. 2d shows that Eq. 3 is a lower bound (hence the “$\simeq$” can be replaced by “$\ge$”); the bound is found to be saturated only when all beads have the same radius, as can be easily proven.^4^

Note that the actual size of the particle does not enter Eq. 3. Once the shape and size are set, the volume of the particle is computed and $r_s$ can be determined. For the given value of *D*, the reference release times $\tau _\alpha ^s$ are then computed by solving Eq. 1 with Eq. 2, and finally the $\tau _\alpha$ is determined. In practice, the particle’s size controls the actual timescale of the release process, while Eq. 3 deals only with relative quantities (ratios) and is unaffected by the size. In the section “Discussion and final remarks,” we will make an example for a particle of linear extension in the millimiter range, i.e., within the conventional range of plastic microparticles (average dimension less than 5 mm).

**Fig. 3:**
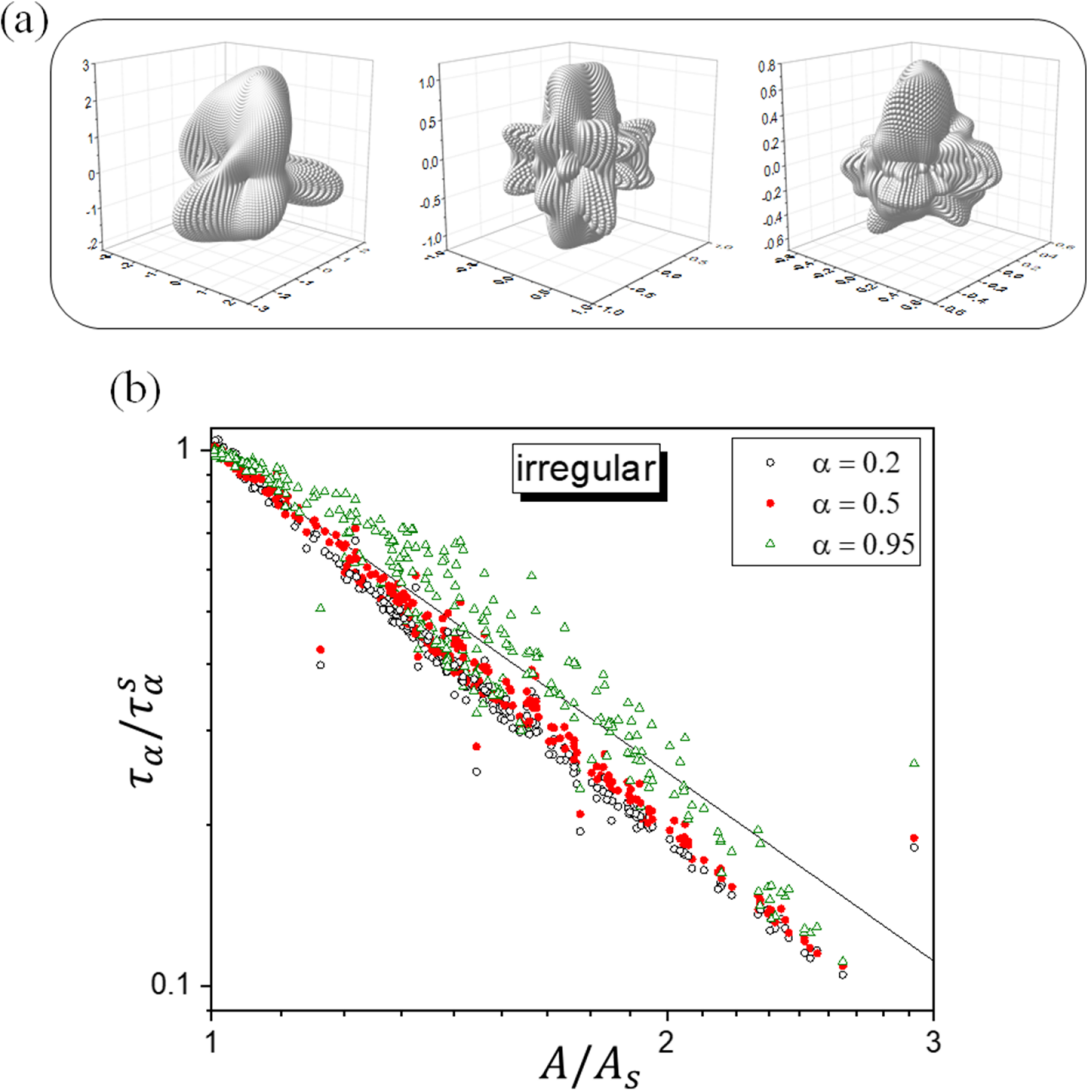
**a** Examples of irregular geometries randomly generated. **b** As for Fig. 2, here for randomly generated instances of irregular geometries (see the text for details)

After emerging from the inspection of regular shapes, Eq. 3 has been tested on randomly generated irregular geometries. The surface of the particles was parametrized in spherical coordinates by choosing, for the radial dependence on the azimuthal and polar angles, a functional form capable of yielding very irregular shapes (see “Computational details”). For the ensemble of produced structures, the ratio between the maximum and the minimum distance from the origin ranged from 1.1 to 270, meaning that the surfaces can feature marked protrusions and deep inlets. Examples of generated structures are shown in Fig. 3a and the results are presented in Fig. 3b. Even in this case, the points fall close to the straight line of slope $-2$, although a more refined exponent ($\simeq -2.4$ in place of $-2$) would be more appropriate for low values of $\alpha$. However, Eq. 3 still captures the trend of $\tau _\alpha / \tau _\alpha ^s$ versus the deformation factor $A/A_s$.

Let us briefly comment Eq. 3 on physical grounds. First we note that it can be rearranged to give

4 $$\begin{aligned} \tau _\alpha \, A^2 \simeq c_\alpha (V) \end{aligned}$$

where $c_\alpha (V)$ depends on the volume (fixed), on *D* and on $\alpha$, but not on the specific shape of the particle. Thus, in principle, one could choose any reference shape for expressing $\tau _\alpha / \tau _\alpha ^\text {ref}$ as in Eq. 3, although the choice of the sphere of equivalent volume seems to be the most natural and convenient one. A simple back-of-the-envelope reasoning can help to rationalize Eq. 4. Let us fix the released fraction $\alpha$ and consider the associated time $\tau _\alpha$. By recalling the root-mean-squared displacement (r.m.s.d) formula for the free Brownian motion, we could take $L_\alpha = \sqrt{2 D \tau _\alpha }$ as the average *linear* distance travelled by the molecules for arriving at the surface in the time $\tau _\alpha$. In doing this, we are consciously making a mistake since while in reality the molecule is meant to remain within the particle, the r.m.s.d. formula is strictly valid only for unrestricted motion. Going anyway forward, let us consider a shell of volume $V_\alpha = \alpha V$ underneath the surface. Still making a “mistake,” we could see $V_\alpha$ as the volume “cleaned” if the molecules initially in it could move only forward towards the surface. For $\alpha$ sufficiently small, we can express such a volume as $V_\alpha = A \, L_\alpha$, hence $L_\alpha = \alpha \, V/A$. By substituting in the r.m.s.d. formula, we get Eq. 4 with a $c_\alpha (V)$ quadratically dependent on $\alpha$ and *V*, and inversely proportional to *D*. Of course, this is nothing but a basic reasoning whose utility is to provide a provisional physical frame of Eqs. 3 and 4. In particular, the reasoning might become critical for featured surfaces and/or for large $\alpha$. This is in accord with the outcomes in Figs. 2 and 3. For the regular geometries, Eq. 3 seems to work well in the first part of the release process (say, for $\alpha$ up to 0.5). For the irregular geometries here explored, the deviations are marked also for small $\alpha$ values, probably in relation with the very featured surfaces. As $\alpha$ increases, tending to the completion of the release, Eq. 3 seems to be followed on average, but the outcomes are more spread with respect to the straight line. Only numerical inspections, like those conducted here, can provide a direct check of the likelihood of Eqs. 3 and 4 case by case.

## Discussion and final remarks

The release kinetics of chemicals from plastic microparticles is of crucial relevance to assess the timescale of pollutants’ inflow in a fluid environment. In this work we have faced the problem of estimating the average times of release, namely the $\tau _\alpha$ where $\alpha$ is the released fraction, for arbitrary shapes of the particles. In fact, while the solution is well-known for spheres, it remains to establish how the release times depend on the specific shape at a fixed volume. The novelties of our approach lie in the fact that (i) generic geometries (even highly irregular) are considered, (ii) the cumbersome numerical solution of the diffusion equation is replaced by the much easier simulation of an ensemble of single-molecule trajectories inside the given microparticle, and (iii) a global analysis of the outcomes is done for extracting general scaling laws of empirical type.

By means of numerical simulations made for some representative categories of regular shapes, and for irregular shapes as well, we have shown that the simple scaling law Eq. 3 can be adopted for estimating $\tau _\alpha$ just in terms of the $\tau _\alpha ^s$ for the equal-volume sphere, and of the ratio $A/A_s$ as a basic descriptor of deformation from the spherical shape.

For some representative regular geometries we have shown that Eq. 3 is accurate, especially if one restricts to the first half of the release process. Let us make an example. Suppose to deal with needle-like particles containing a chemical species with diffusion coefficient $D = 10^{-14} \, \text {m}^2 \text{s}^{-1}$, of the order of the diffusion coefficient of toluene in high-density polyethylene at 25 $^\circ$C (Teuten et al. 2009). Suppose that the particles’ shape can be approximated to cylinders (a shape not among the ones considered above) of length 3 mm and radius 0.1 mm. The radius of the equivalent sphere is 0.28 mm and $\tau _{0.5}^s$, obtained from the profile of $p_\text {int}^s(t)$ in Eq. 2, is equal to 68 h. Given that $A / A_s = 1.944$, by means of Eq. 3 we estimate $\tau _{0.5} = 18 \, \text {h}$, much shorter than the time computed with the spherical approximation. Notably, the exact value from the simulation of an ensemble of Brownian trajectories turns out to be 17.4 h, very close to the above estimate.

We stress again that although Eq. 3 (or, equivalently, Eq. 4) meets the intuitive expectation, the analysis conducted here served to assess its effective applicability to regular geometries that mimic common plastic microfragments (like flakes and needle-like particles) and to irregular shapes. Of course, the full space of the possible shapes cannot be systematically spanned with this empirical approach. The idea is that Eq. 3 can be provisionally used for an initial estimate of the $\tau _\alpha$ and, if the outcome is of particular interest (or concern), the exact computation can then be done for the specific geometry by using the computational tool described in “Computational details.”

Finally, the adoption of the sole ratio $A/A_s$ as a descriptor of the deviation from the spherical shape constitutes the first-level approach. The same kind of analysis could be conducted by adding more descriptors (to be found); again, empirical expressions, more refined than Eq. 3, could be worked out from the distribution of the points $\tau _\alpha / \tau _\alpha ^s$ in such an augmented space of geometrical descriptors. This might be an interesting direction for future inspections.

We want to finish by outlining the limitations of the model, some possible improvements, and the points of strength of the approach. We stress again that our analysis is based on the assumption that the dynamics inside the particle is a diffusive process, which is plausible for amorphous plastics in the rubbery state above the glass transition temperature. It is assumed that the conformational fluctuations of the polymeric structure generate transient voids and create the free volume for the molecules’ motion. In the glassy state, or even in the crystalline state, different kinds of dynamics take place and the model should be revised. Moreover, we took the diffusion coefficient *D* constant inside the particle. For markedly inhomogeneous materials, a location-dependent coefficient should be considered. This can be easily implemented in the simulation of the Brownian trajectories. We also assumed that the water is well mixed in the neighborhood of the particle, so that the concentration at the interface can be taken equal to 0. Although this assumption is commonly done in the modelling of desorption from microparticles (Teuten et al. 2009; Town and van Leeuwen 2020), an improved description should include a stagnant film at the water side, taking into account the partition coefficient plastic-water for the chemical species of interest, and the diffusion in the stagnant film of given thickness. Also this feature can be easily implemented in the simulation route: the forward-backward crossing of the interface plastic-water can be described by means of the Monte Carlo acceptance/rejection criterion (Allen et al. 1987), taking into account that the energy difference between plastic and water is related to the logarithm of the partition coefficient. A more complex scenario is that of plastics with persistent pores at the meso- or even macroscale (widths larger than 50 nm) (Silverstein et al. 2011), either closed or inter-connected. In this situation, the surface enclosing the pores behaves as a reflecting boundary from the side of the plastic. The penetration of water inside the porous material can be likely neglected, especially if considering the most common polyolefins that are found in the aquatic environment (the water absorption is very little^5^ and takes place in a long timescale). In the peculiar case of materials with relevant hydrophilicity, on the contrary, one should consider the possible diffusion of the molecules in two phases, namely the plastic material and the embedded water (Seland and Hafskjold 2001), also including the crossing of the interface plastic-water. Given the geometrical details of a microparticle’s structure, the resulting release kinetics derives from the interplay of all such processes. The simple empirical scaling law obtained in this work is probably violated, and new systematic explorations are required to extend the analysis to such a more complex scenario. While the solution of the diffusion equation would be hardly feasible because of the difficulty of enforcing the specific boundary conditions, the simulation of Brownian trajectories is expected to be a much easier task.^6^ This is a point of strength of the approach presented here.

## Computational details

### Random production of the structures

For each kind of geometry, various instances have been generated by assigning random values to the geometric parameters. Concerning the regular geometries, the parameters are as follows: the lengths of the sides for the parallelepiped, the lengths of the semi-axes for the ellipsoid, the circle’s radius *a* and the revolution radius $R_0 \ge a$ for the torus, the number of beads and their radii for the multi-bead particles. For parallelepiped, ellipsoid, and torus, 200 instances were generated (corresponding to the points in panels a, b, and c of Fig. 2): 100 instances with uniform random drawing of the value of each parameter in the linear scale between 0 and 1, and 100 instances with uniform random drawing in logarithmic scale between $10^{-2}$ and 1. For the multi-bead particles, the number of beads was varied from 2 to 100; for each number of beads, 5 instances were produced by generating the radii at random from the uniform distribution between 0.1 and 1. In total, 495 instances (corresponding to the points in Fig. 2d) were generated.

The surface of the irregular geometries was represented in spherical coordinates $(\theta , \phi , r(\theta ,\phi ))$ with distance from the origin generated as follows. Let $Q(\theta ,\phi ) = \sum _{j=1}^{j_\text {max}} \sum _{m=-j}^{+j} \left[ \text {Re}\{ c_{j,m} Y_{jm}(\theta ,\phi ) \} \right] ^q$, where $Y_{jm}$ are spherical harmonics of rank *j* and projection index *m*, $c_{j,m}$ are complex numbers with real and imaginary parts randomly drawn in the interval $[-c, +c]$ (with *c* fixed, see below), $j_\text {max}$ is the maximum rank (set equal to 5 in all cases), and *q* is an integer exponent which controls the degree of surface irregularity and the extension of the particle. Then we have set $r(\theta ,\phi ) = r_0 + Q(\theta ,\phi ) - \text {min}_{\theta ,\phi }\{ Q(\theta ,\phi ) \}$ where $r_0$ is an offset which corresponds to the minimum distance from the origin. Such a subjective functional form proved useful for producing rather irregular surfaces. In total, 300 instances have been generated (corresponding to the points displayed in Fig. 3b): 67 instances refer to $c = 0.5$ and $q = 1$, 66 instances to $c = 0.5$ and $q = 2$, 67 instances to $c = 0.5$ and $q = 3$, 50 instances to $c = 1$ and $q = 2$, 50 instances to $c = 1$ and $q = 3$. In all cases, the offset $r_0$ was randomly generated between $10^{-2}$ and 1 with uniform distribution in logarithmic scale. By denoting with $r_\text {max}$ and $r_\text {min}$ the maximum and the minimum distances of the surface points from the origin, the ratio $r_\text {max}/r_\text {min}$ varied from 1.10 to 270 for the instances with $c = 0.5$, and from 1.95 to 146 for $c = 1$.

### Surface area and volume

For all the regular shapes, surface area and volume were computed by means of the analytical formulas. For the ellipsoidal particles, the calculation of the surface area was done by using Thomsen’s approximation which is accurate within about 1% and allows avoiding the computation of elliptic integrals.^7^ For the irregular shapes, volume and area were computed by means of numerical integration.^8^

### Survival fractions

For the parallelepiped, the ellipsoid, the torus, and the irregular shapes, the survival fraction $p_\text {int}(t)$ was determined from the simulation of $5\times 10^4$ Brownian trajectories simply counting the fraction of trajectories for which the moving point was still inside the particle. The points shown in Figs. 2 and 3 refer to single sets of simulations, after having established that the variability of the outcomes under repetition of the simulations was very little (see “Accuracy assessment” in the following).

For the reference sphere, the computation of $p_\text {int}^s(t)$ was done by means of Eq. 2 with $n_\text {max} = 100$ components, under check that this was sufficient to achieve a converged profile. For the multi-bead particles made of *N* beads, $p_\text {int}(t)$ was computed as $p_\text {int}(t) = V^{-1} \sum _{i=1}^{N} V_i \, p_{\text {int},i}^s(t)$ with $p_{\text {int},i}^s(t)$ obtained from Eq. 2 for the *i*th bead of volume $V_i$, and $V = \sum _i V_i$ the total volume. In all cases, the times $\tau _\alpha$ were numerically determined from the profiles of the survival fractions.

### Brownian trajectories

For each instance of parallelepiped, elliposid, torus, and irregular shapes, $5\times 10^4$ Brownian trajectories were generated starting from internal points uniformly distributed. Except for the parallelepiped (for which the production of internal points is trivial), the initial points were generated by enclosing the particle in a sphere of radius $R_\text {cut}$ (exactly containing the particle, or slightly greater) and then drawing points at random within the sphere. In spherical coordinates, this requires generating at random three numbers, $u_1$, $u_2$, and $u_3$, from the uniform distribution between 0 and 1,^9^ and then computing $\theta = \arccos (2u_1 -1)$, $\phi = 2 \pi u_2$, $r=R_\text {cut} \root 3 \of {u_3}$. The point is accepted if it falls inside the particle, rejected otherwise.

The Brownian trajectories were generated by means of Langevin’s equation in the overdamped regime of motion (Zwanzig 2001). The evolution rule is $r_j(t+\Delta t) = r_j(t) + s_j \sqrt{2 D \, \Delta t}$, where $r_j(t)$ is the *j*th Cartesian coordinate of the molecule’s position at time *t*, $\Delta t$ is the time step of advancement, *D* the diffusion coefficient, and $s_j$ are random numbers drawn from the Gaussian distribution with null average and unit variance (i.e., Gaussian White Noise was adopted, as typically done for Brownian dynamics^10^ ). The time-step was arbitrarily set equal to $\Delta t = 5 \times 10^{-5} \tau$, where $\tau = r_s^2 / (\pi ^2 D)$ (with $r_s$ the radius of the equal-volume sphere) is taken as a rough estimate of the release timescale. Case by case it was however checked that $\Delta t \le 10^{-2} d_\text {min}^2 / D$ with $d_\text {min}$ a short length of the particle (e.g., the length of the smallest semi-axis of the ellipsoid); this ensures that also the shortest escape trajectories are sufficiently well described. Each trajectory was interrupted when the moving point crossed the delimiting surface.

### Accuracy assessment

The accuracy on $p_\text {int}(t)$ obtained from the Brownian trajectories was assessed by means of the following checks. (A) For a spherical particle, it was checked that $p_\text {int}(t)$ obtained from Brownian trajectories visually matches the exact solution Eq. 2 when the plots are superimposed. In quantitative terms, it was checked that the $\tau _\alpha$ from the simulations, for $\alpha = 0.2, 0.5, 0.95$, were in agreement with the exact values. The deviation $\epsilon = (\tau _\alpha )_\text {sim} / (\tau _\alpha )_\text {exact} - 1$ was adopted for such a check. The simulations were repeated 100 times. The distribution of the $\epsilon$ values showed a median of $+0.050$ for $\alpha = 0.2$ (the maximum displacement from 0 was $+0.10$), $+0.012$ for $\alpha = 0.5$ (maximum displacement $+0.037$), and $-0.0004$ for $\alpha = 0.95$ (maximum displacement $-0.022$). (B) For some of the irregular geometries, it was checked the stability of the ratio $\tau _{0.5}/\tau _{0.5}^s$ both under the repetition of the simulations^11^ and with respect to the number of trajectories. For instance, for the first irregular geometry generated with $c = 0.5$ and $q = 1$, the variability of the outcomes with $5 \times 10^{4}$ trajectories was found of 0.9% (expressed as standard deviation over mean value from 10 repetitions). Concerning the convergence with respect to the number of trajectories, for the same geometry it was found that the outcome is stable already with $10^4$ trajectories, and that the fluctuations of the outcomes are of the order of 1% in passing from $10^4$ to $10^6$ trajectories. In summary, both the method for sampling the starting points and the number of $5 \times 10^4$ trajectories for each considered particle can be considered adequate for the present analysis.

## Data Availability

All data generated and analyzed during this study are displayed in the article.

## Footnotes

1 Although our focus is on the release of additives, microplastics can absorb (and hence transport) pollutants in the aquatic environment. For a list of typical absorbed pollutants, see Table 2 of Ref. Amelia et al. (2021) and references therein. We can find hydrophobic organic species like polycyclic aromatic hydrocarbons (PAH) and polychlorinated biphenyl (PCB), hydrophilic species like perfluoroalkyl acids, heavy metals, and drugs.

2 For data on the glass transition temperature of synthetic polymers, see for instance PerkinElmer 2019, “Melting point, glass transition temperature and structure of common polymers” (available at https://resources.perkinelmer.com/corporate/cmsresources/images/44-74863tch_mptgandstructureofcommonpolymers.pdf), and Omnexus - The material selection platform (available at https://omnexus.specialchem.com/polymer-properties/properties/glass-transition-temperature).

3 All simulations and post-production analyses were done with Fortran77 codes developed by implementing the route and the choices fully described in “Computational details.”

4 For *N* identical beads, we have $(r_i/r_s)=N^{-3}$ (from the equal-volume condition) and $A/A_s = N (r_i/r_s)^2$. Thus, it must be $r_i/r_s = A_s / A$. For identical beads, the $\tau _\alpha$ for the whole multi-bead particle and for the single bead do coincide. By considering the equal-volume sphere on one side, and the single bead on the other side, from Eq. 2 it can be seen that the same fraction of release is achieved if $\tau _\alpha ^s/r_s^2 = \tau _\alpha / r_i^2$. By using the above result, it follows that $\tau _\alpha = \tau _\alpha ^s (A/A_s)^2$ in this peculiar case.

5 Data on water absorption of several plastics can be found at https://omnexus.specialchem.com/polymer-properties/properties/water-absorption-24-hours.

6 Let us sketch the possible route for the general case of hydrophilic material and presence of water inside the particle. While the molecule is in one of the two phases, the motion is described by the Langevin equation with the specific diffusion coefficient. The reflecting boundaries are easily implemented by checking when a move would take the molecule to the other side, and then, for instance, making a simple reflection of the forbidden part of the move with respect to the tangent plane to the boundary at the intersection point. The crossing of the interface plastic-water could be handled by means of the Monte Carlo acceptance/rejection criterion (Allen et al. 1987). The trajectories have to start from points sampled from a reasonable initial distribution (for instance, homogeneous distribution in the plastic). From an ensemble of trajectories, one can obtain $p_\text {int}(t)$ (once the outer surface that separates the particle from the environment is specified), and hence characterize the release kinetics.

7 The approximation due to Knud Thomsen can be found at https://www.numericana.com/answer/ellipsoid.htm#thomsen.

8 The numerical integrations were done with the double-precision IMSL® Fortran77 subroutine ‘DQAND.f’(http://www.roguewave.com/products/imslnumerical-libraries.aspx); absolute and relative accuracy were set equal to $10^{-4}$ and $5 \times 10^{-3}$, respectively.

9 The uniform deviates in [0, 1] were generated by means of the basic “Linear Congruential Generator” employed in “Computer Simulation of Liquids” by M. P. Allen and D. J. Tildesley (Allen et al. 1987); see the Fortran77 function “ranf” in the program F.11 available at http://www.ccp5.ac.uk/software/allen_tildersley. It was checked that equivalent results are obtained by using the more elaborated generator implemented in the function “ran2.f” of “Numerical Recipes in FORTRAN 77” (Press et al. 1992).

10 The Normal deviates were generated with the double-precision Fortran77 function “drnorf.f” available, for instance, at https://gams.nist.gov/cgi-bin/serve.cgi/Module/NMS/DRNOR/11308/

11 The variability is due to the fact that the initial points are randomly generated and the trajectories are stochastic. The independence of the simulations was ensured by using, for each repetition, different seeds for the random number generators (the seeds were manually initialized for the generation of the initial points, and initialized with the computer’s clock for the Brownian trajectories).
