# Correction to: Shape dependence of the release rate of chemicals from plastic microparticles

**DOI:** 10.1007/s11356-022-22104-x

**Published:** 2022-07-20

**Authors:** Riccardo Frazzetto, Diego Frezzato

**Affiliations:** Department of Chemical Sciences, University of Padova, via Marzolo 1, I-35131 Padova, Italy

**Correction to: Environmental Science and Pollution Research**

10.1007/s11356-022-21440-2

In page 7, section Computational details, Random production of the structures, the text in the 12^th^ line "10^{-2}" should be "10^−2^".

The Original article has been corrected.

